# Use of a Baculovirus-Mammalian Cell Expression-System for Expression of Drug-Metabolizing Enzymes: Optimization of Infection With a Focus on Cytochrome P450 3A4

**DOI:** 10.3389/fphar.2022.832931

**Published:** 2022-02-22

**Authors:** Yuu Miyauchi, Akane Kimura, Madoka Sawai, Keiko Fujimoto, Yuko Hirota, Yoshitaka Tanaka, Shinji Takechi, Peter I. Mackenzie, Yuji Ishii

**Affiliations:** ^1^ Laboratory of Hygienic Chemistry, Faculty of Pharmaceutical Sciences, Sojo University, Kumamoto, Japan; ^2^ Division of Pharmaceutical Cell Biology, Graduate School of Pharmaceutical Sciences, Kyushu University, Fukuoka, Japan; ^3^ Clinical Pharmacology, College of Medicine and Public Health, Flinders Medical Centre and Flinders University, Adelaide, SA, Australia

**Keywords:** drug-metabolizing enzyme, heterologous expression, baculovirus, mammalian cell, bac-mam system, cytochrome P450 3A4, UDP-glucuronosyltransferase

## Abstract

Heterologous expression systems are important for analyzing the effects of genetic factors including single nucleotide polymorphisms on the functions of drug-metabolizing enzymes. In this study, we focused on a baculovirus-mammalian cell (Bac-Mam) expression system as a safer and more efficient approach for this purpose. The baculovirus-insect cell expression system is widely utilized in large-scale protein expression. Baculovirus has been shown to also infect certain mammalian cells, although the virus only replicates in insect cells. With this knowledge, baculovirus is now being applied in a mammalian expression system called the Bac-Mam system wherein a gene-modified baculovirus is used whose promotor is replaced with one that can function in mammalian cells. We subcloned open-reading frames of cytochrome P450 3A4 (CYP3A4), UDP-glucuronosyltransferase (UGT) 1A1, and UGT2B7 into a transfer plasmid for the Bac-Mam system, and prepared recombinant Bac-Mam virus. The obtained virus was amplified in insect Sf9 cells and used to infect mammalian COS-1 cells. Expression of CYP3A4, UGT1A1, and UGT2B7 in COS-1 cell homogenates were confirmed by immunoblotting. Optimum infection conditions including the amount of Bac-Mam virus, culture days before collection, and concentration of sodium butyrate, an enhancer of viral-transduction were determined by monitoring CYP3A4 expression. Expressed CYP3A4 showed appropriate activity without supplying hemin/5-aminolevulinic acid or co-expressing with NADPH-cytochrome P450 reductase. Further, we compared gene transfer efficiency between the Bac-Mam system and an established method using recombinant plasmid and transfection reagent. Our results indicate that the Bac-Mam system can be applied to introduce drug-metabolizing enzyme genes into mammalian cells that are widely used in drug metabolism research. The expressed enzymes are expected to undergo appropriate post-translational modification as they are in mammalian bodies. The Bac-Mam system may thus accelerate pharmacogenetics and pharmacogenomics research.

## Introduction

Hydrophobic xenobiotics are difficult to be eliminated and induce adverse effects in the body for a long period. Drug-metabolizing enzymes constitute a defensive mechanism against such chemicals by converting them to less toxic and soluble metabolites, although pharmacologically active or toxic reactive metabolites of drugs are occasionally formed. Hence drug metabolizing enzymes are important for both the detoxication and intoxication of drugs (Guengerich, 2006). Large inter-individual differences in drug metabolism capacity are largely due to genetic and environmental factors. Single nucleotide polymorphisms play a major role among genetic factors (Evans and Relling, 1999). Cytochrome P450 (P450, CYP) and UDP-glucuronosyltransferase (UGT) play crucial roles in respective phase I and II reactions of drug metabolism. Huge numbers of genetic polymorphisms were revealed which can affect functions of P450 and UGT, with some causing adverse effects to drugs (Court, 2010; Sugatani, 2013; Zanger and Schwab, 2013). Hence, it is important to elucidate whether newly observed genetic polymorphisms affect activities of drug-metabolizing enzymes using a simple approach.

An heterologous expression system is an essential approach to address this issue. Actually, many systems have been applied to research of drug metabolism (Guengerich et al., 1997; Crespi and Miller, 1999). Among them, expression systems using mammalian cells are the most physiological since expressed protein is expected to undergo post-translational modification, locate to the correct target organelle, and show proper activity. There are several ways to introduce a target gene into mammalian cells, including transfection of recombinant plasmid with calcium phosphate, chemical reagent, or electroporation and use of recombinant adeno- or other viruses (Khan, 2013). These methods have both advantages and disadvantages. In this study, we focused on a baculovirus-mammalian cell (Bac-Mam) expression system as a safe and efficient method to express introduced genes. Baculovirus was originally utilized for transient expression of protein in insect cells (Luckow, 1993). Because the baculovirus host is restricted to insect cells, the baculovirus-insect cell expression system is considered as a safe expression system without negative impacts on animals (Hu, 2005). Of course, other viral vectors also lack inherent ability to replicate freely in mammalian cells but experimental restrictions in facility and equipment were less in the case of baculovirus. Despite the restricted host, it was reported that baculovirus can infect human cells (Volkman and Goldsmith, 1983). Focusing on this newly observed infection ability, a Bac-Mam system was established in which a promoter-substituted baculovirus, Bac-Mam virus, is generated for expression in mammalian cells (Hu, 2005; Morales-Perez et al., 2016). The Bac-Mam system is considered safer than other virus-used expression systems because the Bac-Mam virus can infect many kinds of mammalian cells but never replicates in these cells. The virus is now widely utilized for gene/siRNA delivery *in vitro* and *in vivo* (Hu, 2005; Makkonen et al., 2015).

In the present study, we applied the Bac-Mam system to transient expressions of major human isoforms of P450 and UGT, namely CYP3A4, UGT1A1, and UGT2B7, in COS-1 cells. We determined infection conditions by monitoring CYP3A4 expression. Further, expression levels were compared: 1) between the Bac-Mam system and transfection of plasmid with a chemical reagent and 2) among several kinds of cells.

## Materials and Methods

### Materials

Synthetic oligonucleotides were purchased from Fasmac (Kanagawa, Japan). Restriction enzymes and shrimp alkaline phosphatase were from Takara Bio (Shiga, Japan). D-luciferin potassium salt was from Nacalai Tesque (Kyoto, Japan) and the reduced form of nicotinamide adenine dinucleotide phosphate (NADPH) was from Oriental Yeast (Tokyo, Japan). Pooled human liver microsomes (HLM) prepared 50 donors, and Supersomes™ expressing CYP3A4 and NADPH-P450 reductase (CPR) were from BD Gentest (Franklin Lakes, NJ, United States). All other reagents were of the highest quality commercially available.

### Subcloning of Drug-Metabolizing Enzyme Genes Into a Bac-Mam and Mammalian Cell Expression Vector

pEZT-BM, a transfer plasmid for the Bac-Mam system, was a gift from Ryan Hibbs (Addgene plasmid # 74099; http://n2t.net/addgene:74099; RRID: Addgene_74099). The plasmid was based on pFastBac Dual (Thermo Fisher Scientific, Waltham, MA, United States), and the original insect polyhedrin promotor was replaced by a cytomegalovirus (CMV) promotor for expression of target gene in mammalian cells (Morales-Perez et al., 2016). The open reading frames (ORFs) of CYP3A4, UGT1A1, and UGT2B7 were amplified with each pair of primers as listed in Supplemental Table S1, and each pFastBac1-based construct as template (Ishii et al., 2014; Miyauchi et al., 2015). KOD-Plus-Neo DNA Polymerase (Toyobo Life Science, Osaka, Japan) was used for the polymerase chain reaction, and thermal cycling condition was as follows: initial denaturation, 94°C, 2 min; 40×amplification step, 98°C, 10 s; 52°C, 30 s; 68°C, 1 min; hold, 4°C. The PCR products were digested with DpnI, purified with a FastGene Gel/PCR Extraction Kit (NIPPON Genetics, Tokyo, Japan), and introduced into KpnI/XhoI digested pEZT-BM using an In-Fusion HD Cloning Kit (Takara Bio). The ORF of CYP3A4 was also subcloned into pcDNA3.1/Hygro (−), a mammalian cell expression vector, with KpnI/XhoI sites using Ligation high Ver.2 (Toyobo Life Science). Primers for this CYP3A4 subcloning are also listed in Supplemental Table S1. The nucleotide sequences of the constructs were confirmed by an Applied Biosystems 3130xl Genetic Analyzer (Thermo Fisher Scientific), using a BigDye Terminator version 3.1 Cycle Sequencing Kit (Thermo Fisher Scientific).

### Preparation of Recombinant Bac-Mam Virus and Culture of Insect Cells

Recombinant Bac-Mam viruses were prepared with the Bac-to-Bac Baculovirus Expression system (Thermo Fisher Scientific). The pEZT-BM constructs were transfected into competent *Escherichia coli* (*E. coli*) DH10Bac. A positive single clone was selected after blue/white colony selection, and recombinant bacmid, a part of the baculoviral DNA, was prepared according to the user manual. Insect cells Sf9 were cultured in a 100 or 500 mL plastic Erlenmeyer flask (Corning, Lowell, MA, United States) containing Sf-900 III medium (Thermo Fisher Scientific) supplemented with 5% fetal bovine serum (FBS). To prepare recombinant Bac-Mam virus, the constructed bacmids were transfected into Sf9 cells as described previously (Miyauchi et al., 2015). The control bacmid, obtained from transfection of mock pEZT-BM, was also transfected to obtain control virus. pEZT-BM contains the p10 promotor followed by the ORF of green fluorescent protein (GFP), which enabled us to confirm viral generation in Sf9 cells by detecting GFP (Supplemental Figure S1). Culture media were collected as primary viruses 1 week after transfection. Baculoviral DNA were purified from portions of these media (200 μL) by NucleoSpin Blood (Macherey-Nagel, Düren, Germany), and their titer was determined using a BacPAK qPCR Titration Kit (Clontech, Mountain View, CA, United States). The Bac-Mam viruses were amplified 2–3 times until their titer reached over 5.0×10^7^ plaque-forming unit (PFU)/mL.

### Culture of Mammalian Cells and Infection of Bac-Mam Virus

In this study, COS-1 cells were mainly utilized for expression of drug-metabolizing genes. COS cells (COS-1 cells, COS-3 cells, and COS-7 cells) were established by transformation of African monkey kidney cells (CV-1 cells) with a mutant of simian virus 40 (SV_40_) (Gluzman, 1981) and have been widely used in research including the field of drug metabolism (Mackenzie, 1986; Clark and Waterman, 1991; Guengerich et al., 1997; Thomae et al., 2002). In case of P450, it is especially advantageous that COS cells have endogenous CPR, which is sufficient for catalytic activity of transiently expressed P450 (Clark and Waterman, 1991). COS-1, HEK293, and HepG2 cells were grown in Dulbecco’s modified Eagle medium (FUJIFILM Wako Pure Chemical, Osaka, Japan, Cat#043-30085) supplemented with 10% FBS. Cells were routinely maintained in a 10 cm dish and seeded to a 35 mm dish at 1.0×10^6^ cells/dish the day before infection. After removing the culture medium, recombinant Bac-Mam viruses were added to the dish. Viral amount, multiplicity of infection (MOI), ranged from 25 to 150 and finally fixed at 100. The infection was performed in a CO_2_ incubator for 1 hour. Then virus was removed, and new culture medium containing sodium butyrate ranged from 0 to 4.5 mM (finally fixed at 3 mM) was added. Sodium butyrate is used as an inhibitor of histone deacetylase, enhancing expression of recombinant protein in mammalian cells including the Bac-Mam system (Gorman et al., 1983; Condreay et al., 1999; Morales-Perez et al., 2016). The infected cells were further cultured for 24–96 h. The culture time was finally fixed at 48 h.

### Plasmid Transfection to COS-1 Cells

COS-1 cells were seeded in a 35 mm dish the day before transfection. Transfection of pcDNA3.1/Hygro (−)_CYP3A4 was conducted as described previously (Miyauchi et al., 2020b). In short, 2 μg plasmid DNA was mixed with 8 μg of Polyethylenimine HCl MAX (PEI; Polysciences, Warrington, PA, United States) in Opti-MEM I Reduced Serum Media (Thermo Fisher Scientific) and incubated at room temperature for 10 min. The DNA-PEI complex was added to COS-1 cells, and the cells were cultured for 48 h.

### Preparation of Cell Homogenates

Cells were collected in homogenate buffer containing 10 mM Tris-HCl (pH7.4), 250 mM sucrose, and 10% glycerol, and then sonicated with an AS38A ultrasonic cleaner (AS ONE, Osaka, Japan). Protein concentration was determined using Quick Start Bradford 1×Dye Reagent (Bio-Rad Laboratories, Hercules, CA, United States) with bovine serum albumin as a standard.

### Immunoblotting

Cell homogenates (20 μg) were separated by sodium dodecyl sulfate-polyacrylamide gel electrophoresis and electroblotted onto a polyvinylidene difluoride membrane (FUJIFILM Wako Pure Chemical, Cat#034-25663). The blots were washed with Tris-buffered saline containing 0.1% Tween 20 (TBS-T) and incubated in TBS-T containing 5% skim milk at room temperature for 30 min. After blocking, the blots were incubated with primary antibody diluted 2,000-fold with TBS-T containing 5% skim milk at 4°C overnight. The following primary antibodies were utilized: mouse anti-CYP3A4 antibody, rabbit anti-UGT1A antibody (respective Cat# sc-53850 and sc-25847; Santa Cruz Biotechnology, Dallas, TX, United States), rabbit anti-UGT2B7 antibody, and rabbit anti-glyceraldehyde-3-phosphate dehydrogenase (GAPDH) antibody (respective Cat# 16661-1-AP and 10494-1-AP, Proteintech, Rosemont, IL, United States). The blots were extensively washed with TBS-T and further incubated with secondary antibody diluted 10,000-fold with TBS-T containing 5% skim milk at room temperature for 1 hour. The following secondary antibodies labeled with horseradish peroxidase (HRP) were utilized: HRP-goat anti-mouse IgG (H + L) antibody and HRP-goat anti-rabbit IgG (H + L) antibody (respective Cat# SA00001-1 and SA00001-2, Proteintech). After extensive washing with TBS-T, EzWestLumi plus (ATTO, Tokyo, Japan) was used as a substrate of HRP, and the signal was visualized and analyzed using iBright Imaging System (Thermo Fisher Scientific). To quantify expressed CYP3A4 in 20 μg homogenates, Supersomes™ (BD Gentest) containing 1 pmol CYP3A4 were applied as a standard.

### CYP3A4 Assay

CYP3A4 activity was measured with P450-Glo CYP3A4 Assay and Screening System (Luciferin-IPA; Promega, Madison, WI, United States). We used 100 μM NADPH (final concentration). Substrate luciferin-IPA concentration varied from 0.3 to 15 μM (nine points of concentration). Chemiluminescence was measured using a microplate reader, Infinite M200 Pro (Tecan Group, Männedorf, Switzerland). As enzyme sources, 20 μg HLM, Supersomes™, or COS-1 homogenates were utilized. In kinetic experiments, data were fitted to the Michaelis-Menten model defined by the equation below:

$$V=\frac{Vmax\times S}{Km+S}$$

Where V is the reaction rate, S is the substrate concentration, Vmax is the maximum enzyme velocity, and Km is the Michaelis constant, which is equal to the concentration of substrate for half-maximal velocity. The kinetic parameters were determined with GraphPad Prism 5.04 software (GraphPad software, La Jolla, CA, United States).

## Results

### Detection of CYP3A4, UGT1A1, and UGT2B7 Expressed With the Bac-Mam System

**FIGURE 1:**
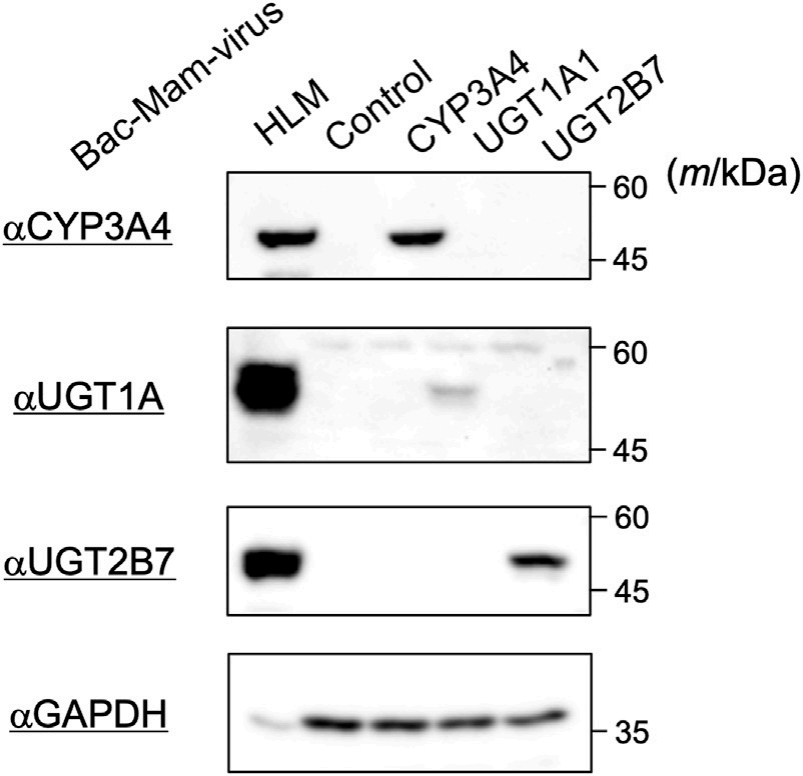
Detection of drug-metabolizing enzymes expressed by the Bac-Mam system. COS-1 cells were infected with the Bac-Mam viruses encoding CYP3A4, UGT1A1, or UGT2B7. Infection conditions were: virus amount, MOI = 100; culture time after infection, 24 h; sodium butyrate, 3 mM. Cell homogenates (20 μg) were analyzed by immunoblotting with HLM (10 μg) and homogenates from cells infected with control virus as positive and negative controls, respectively. HLM, human liver microsomes.

The drug-metabolizing enzymes were transiently expressed using the Bac-Mam system in COS-1 cells and their expressions were confirmed by immunoblotting (Figure 1). Infection conditions were fixed as below: MOI = 100; culture time, 24 h; sodium butyrate, 3 mM. With every enzyme, specific bands were observed that were not detectable in control virus-infected cells. In addition, the enzymes showed the same mobility as those in the positive control, HLM. The antibody that we used in detection of UGT1A1 can react with other UGT1A isoforms in HLM, which resulted in a slight difference of observed bands between HLM and COS-1 homogenates. These results indicated that the Bac-Mac system is suitable for transient expression of the major drug-metabolizing enzymes. In this experiment, we loaded 20 μg homogenates and 10 μg HLM. CYP3A4 showed the closest expression to that in HLM among the three targets, so we optimized infection conditions by monitoring CYP3A4 expression level in subsequent experiments.

### Optimization of Viral Amount

**FIGURE 2:**
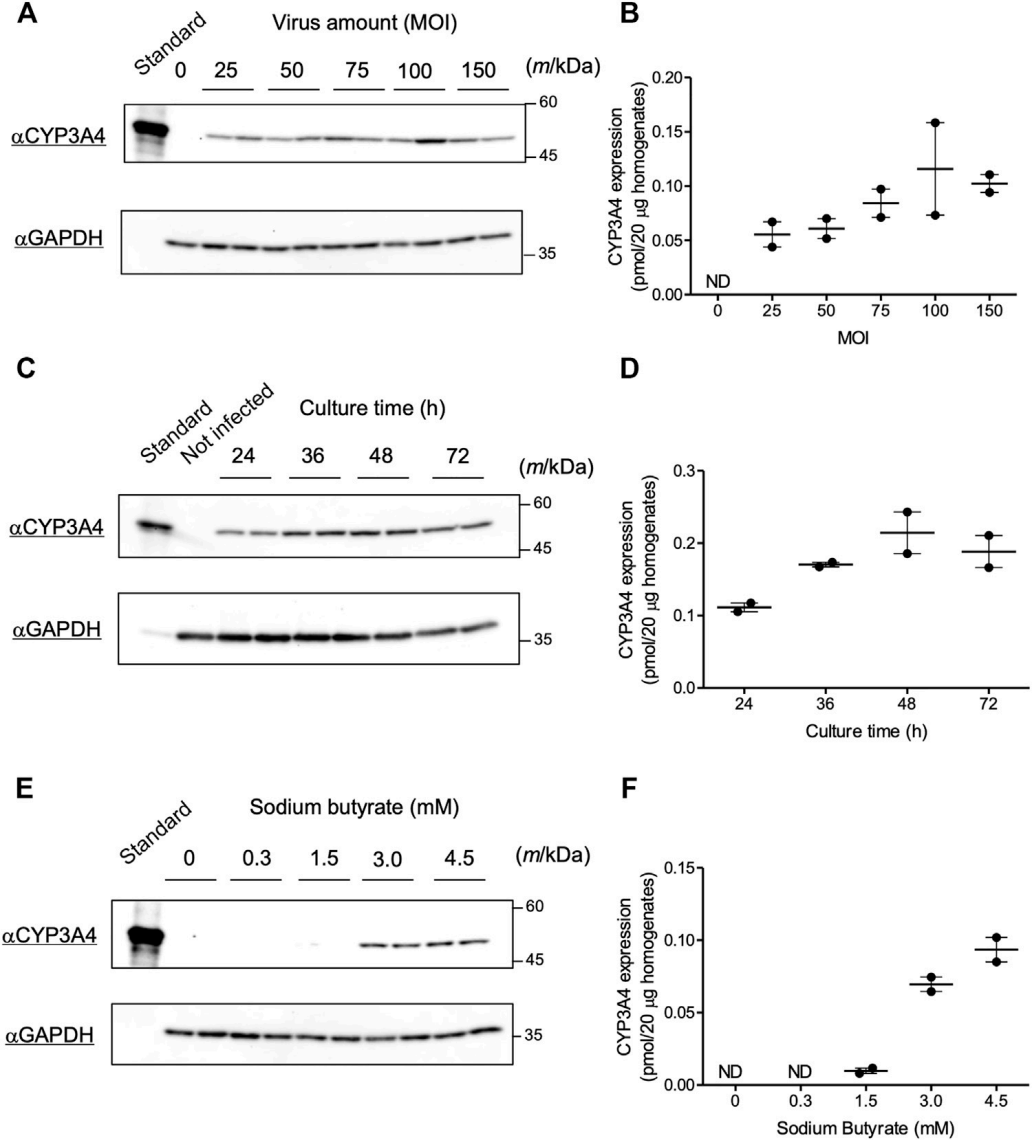
Optimization of infection conditions for the Bac-Mam virus. Conditions for the Bac-Mam virus to infect COS-1 cells were optimized by monitoring CYP3A4 expression. CYP3A4 expression in COS-1 homogenates (20 μg) was determined by immunoblotting with Supersomes™ contained 1 pmol CYP3A4 (BD Gentest) as a standard. Viral infection was independently conducted twice in each experiment. **(A)** Optimization of viral amount (MOI) ranging from 0 to 150. Culture time after infection and concentration of sodium butyrate were fixed at 24 h and 3 mM, respectively. **(B)** The result of quantification of CYP3A4 in the optimization of viral amount. Each determined CYP3A4 level (N = 2), and the mean ± S.E.M. are presented. **(C)** Optimization of culture time after infection ranging from 24 to 72 h. Viral amount and concentration of sodium butyrate were fixed at MOI = 100 and 3 mM, respectively. **(D)** The result of quantification of CYP3A4 in the optimization of culture time. Each determined CYP3A4 level (N = 2), and the mean ± S.E.M. are presented. **(E)** Optimization of the concentration of sodium butyrate ranging from 0 to 4.5 mM. Viral amount and culture time after infection were fixed at MOI = 100 and 48 h, respectively. **(F)** The result of quantification of CYP3A4 in the optimization of concentration of sodium butyrate. Each determined CYP3A4 level (N = 2), and the mean ± S.E.M. are presented. ND, not detected.

We evaluated infection conditions to obtain maximum CYP3A4 expression using the recombinant Bac-Mam virus in COS-1 cells. P450 can be quantified by measuring its CO difference spectrum (Omura and Sato, 1964), but a previous study suggested that the amount of P450 that can be overexpressed in COS-1 cells was below the amount required for detection using the CO difference spectrum assay (Clark and Waterman, 1991). Therefore, we quantified expressed CYP3A4 by immunoblotting in the current study. First, we tested varying the amount of virus. In our previous study, CYP3A4, CPR, and some UGT isoforms were expressed in insect Sf9 cells using an original baculovirus expression system. The viral amounts in the experiments were set at MOI = 0.01–1, which was enough to express the enzymes in Sf9 cells (Ishii et al., 2014; Miyauchi et al., 2015; Miyauchi et al., 2020a). In contrast, more than 10-fold larger amounts of virus seem to be necessary in the Bac-Mam system since the Bac-Mam virus cannot amplify in mammalian cells, and so secondary infection is not expected (Condreay et al., 1999). Hence, COS-1 cells were infected with the prepared Bac-Mam virus at MOI = 0 (not infected), 25, 50, 75, 100, and 150. Culture time and concentration of sodium butyrate were fixed at 24 h and 3 mM, respectively. Immunoblotting and its analyzed result are shown in Figures 2A,B, respectively. CYP3A4 expression tended to increase in a MOI-dependent manner and reach a peak around MOI = 75–100. Hence, MOI was fixed at 100 in the later experiments.

### Optimization of Culture Time After Infection

Next, we tested varying culture time after viral infection. Viral amount and concentration of sodium butyrate were fixed at MOI = 100 and 3 mM, respectively. Bac-Mam virus-infected cells began to float from dishes 48 h after infection, and almost all the cells were dead at 96 h. Thus, we prepared cell homogenates between 24 and 72 h post infection and quantified CYP3A4 expressions (Figures 2C,D). Expression levels increased in a time-dependent manner and reached a maximum at 48–72 h after infection. Given that the cells began to die, we concluded that 48 h is the best culture time after infection.

### Optimization of Concentration of Sodium Butyrate

As cell death was also observed in the absence of viral infection, we predicted that sodium butyrate was the cause of this toxicity. We next varied the concentration of sodium butyrate from 0 to 4.5 mM under the fixed condition: viral amount, MOI = 100; culture time, 48 h. As explained, sodium butyrate enhances target gene expression in the Bac-Mam system. Hence, CYP3A4 was not detectable under 1.5 mM while significant expression was observed at three and 4.5 mM (Figures 2E,F). In accordance with our hypothesis, however, not only significant CYP3A4 expression but also cell toxicity was observed, and COS-1 cells seemed to be more sensitive to sodium butyrate than HEK293 and HepG2 cells. We determined that 3 mM is the best concentration of sodium butyrate for use. Taken together, we concluded that the optimized infection condition was: viral amount, MOI = 100; culture time after infection, 48 h; concentration of sodium butyrate, 3 mM.

### Comparison of CYP3A4 Activity With HLM and Supersomes™

**FIGURE 3:**
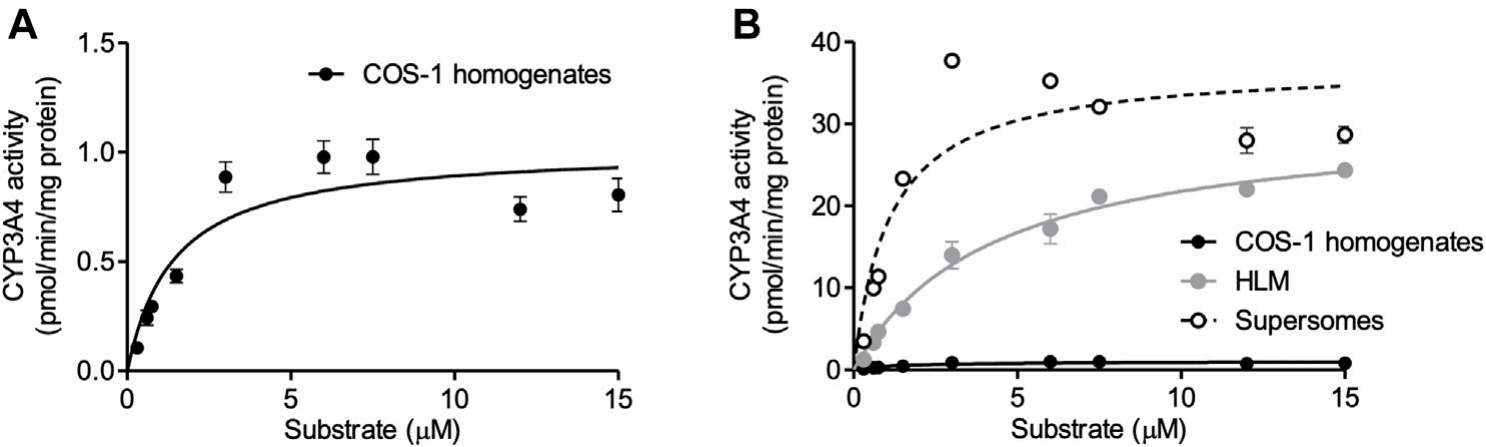
Kinetic analysis of expressed CYP3A4 with the Bac-Mam system. Kinetic analysis of CYP3A4 was conducted with luciferin-IPA (Promega) as a substrate. **(A)** COS-1 homogenates (20 μg) were used as enzyme source, and the data were fitted to the Michaelis-Menten equation. Results are shown as the mean ± S.E.M. of three independent transductions. **(B)** Comparison of CYP3A4 activity between prepared COS-1 homogenates and HLM/Supersomes™. The result of kinetics with HLM/Supersomes™ (20 μg) as enzyme source were merged. In the result of HLM and Supersomes™, each plot represents the mean ± S.E.M. of triplicate assays. Calculated parameters were listed and compared in the Table.

According to the optimized infection condition, COS-1 homogenates were prepared, and the activity of expressed CYP3A4 was measured with luciferin-IPA as a substrate, which is converted to luciferin by CYP3A4 but shows minimal cross-reactivity with CYP3A5 and CYP3A7. Kinetic analysis was conducted with the COS-1 homogenates, HLM, and Supersomes™ as enzyme sources, and obtained parameters were compared (Figure 3 and Table 1). CYP3A4 activity was confirmed in the prepared homogenates, which supported our view that the Bac-Mam system can be applied for expression of drug-metabolizing enzymes. The highest CYP3A4 activity was observed when Supersomes™ was used as the enzyme source, followed by HLM and homogenates. In comparison with HLM and Supersomes™, the Vmax value of the Bac-Mam expressed CYP was one 30th of those of HLM/Supersomes™ although the Km value was comparable.

**TABLE 1:** Comparison of kinetic parameters of CYP3A4 activity among COS-1 cell homogenates prepared by the Bac-Mam system, HLM, and Supersomes™.

	Vmax (pmol/min/mg protein)	Km (μM)
COS-1 homogenates	1.0 ± 0.1	1.5 ± 0.4
HLM	31.0 ± 1.6	4.3 ± 0.6
Supersomes™	37.2 ± 2.8	1.1 ± 0.3

Data were fitted to the Michaelis-Menten equation (Figures 3A,B). Each parameter is shown as the calculated value ±S.E.M. HLM, human liver microsomes.

### Comparison of Bac-Mam Expressed CYP3A4 Levels With Plasmid Expressed Levels

**FIGURE 4:**
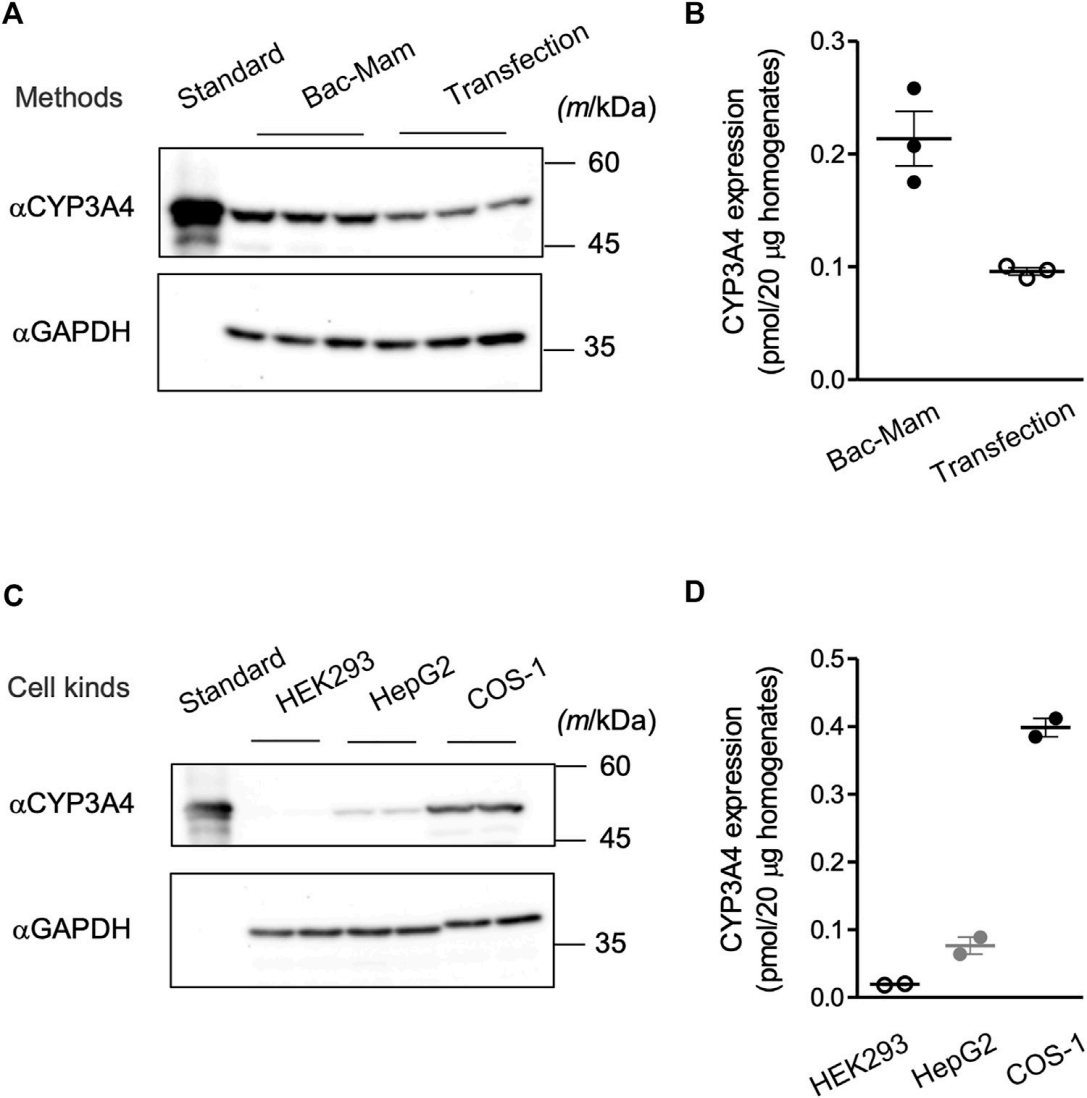
Advantages of the Bac-Mam system. **(A)** CYP3A4 expression levels were compared between the Bac-Mam system and plasmid transfection with polyethylenimine (PEI). Viral infection and transfection were independently conducted three times, and COS-1 cell homogenates (20 μg) were analyzed by immunoblotting with Supersomes™ (BD Gentest) containing 1 pmol CYP3A4 as a standard. **(B)** The result of quantification of expressed CYP3A4 in the comparison between the two expression systems. Each determined CYP3A4 level (N = 3), and the mean ± S.E.M. are presented. **(C)** Transduction efficiency of Bac-Mam virus was compared among three kinds of mammalian cells. Viral infection conditions were: viral amount, MOI = 100; culture time after infection, 48 h; concentration of sodium butyrate, 3 mM. Cell homogenates (20 μg) were applied to determine the expressed CYP3A4 level. Infection was independently conducted twice. **(D)** The result of quantification of CYP3A4 expressed in the three kinds of cells. Each determined CYP3A4 level (N = 2), and the mean ± S.E.M. are presented.

Transfection of recombinant plasmid with a transfection reagent is one of the major methods to introduce a target gene into mammalian cells and is also utilized in research of drug-metabolizing enzymes. We compared the expressed CYP3A4 levels between the Bac-Mam system and the widely used transfection method (Figures 4A,B). With the Bac-Mam system, 2–3 times higher expression of CYP3A4 was observed compared to that expressed using the transfection method.

### Application of the Bac-Mam System to Other Kinds of Mammalian Cells

We have applied the Bac-Mam system to introduce genes of CYP3A4 and some UGTs into COS-1 cells and confirmed their expression and CYP3A4 function. Further, we utilized the Bac-Mam system to introducing CYP3A4 into other kinds of cells (HEK293 and HepG2 cells) under the optimized condition determined with COS-1 cells. Although these cells are widely used in research of drug-metabolizing enzymes, there was a large difference in the expressed CYP3A4 levels (Figures 4C,D). COS-1 cells showed the highest CYP3A4 level, almost 4- and 20-times higher than that in HepG2 and HEK293 cells, respectively.

## Discussion

In this study, we have applied a Bac-Mam system to introduce genes of drug-metabolizing enzymes into COS-1 cells and optimized infection conditions focusing on CYP3A4. The optimized viral amount was determined at MOI = 100 (Figures 2A,B), which is over 100 times higher compared to that of the baculovirus-insect cell expression system (Miyauchi et al., 2015). This is because the Bac-Mam virus never replicates in mammalian cells, so there is no secondary infection. However, the viral amount is comparable to the amount of adenovirus needed for maximum CYP3A4 expression in HepG2 cells (Aoyama et al., 2009) and those of Bac-Mam virus in other studies (Condreay et al., 1999; Ramos et al., 2002). Since it is easy to culture insect cells in large scale, such a requirement for a high amount of virus does not matter. Culture time after infection was optimized at 48 h (Figures 2C,D), but caution is required to minimize sodium butyrate induced cellular toxicity. As shown in Figures 2E,F, sodium butyrate was essential for expression of CYP3A4 but COS-1 cells seemed to be sensitive to this chemical. Sodium butyrate has many cellular effects including inhibition of histone deacetylase, e.g. arresting cell proliferation, affecting the cytoskeleton, and sometimes triggering apoptosis (Kruh, 1982; Shin et al., 2012). The main cause of COS-1 death remains unclear but further medium replacement should be carried out to reduce toxicity when experiments on Bac-Mam virus-infected cells require several days of culture.

Since mammalian cells were utilized in this study, it was not necessary to supply hemin and 5-aminolevulinic acid which are essential for holo-CYP3A4 enzyme formation in insect cells and *E. coli* (Asseffa et al., 1989; Kusano et al., 1999). Neither was coexpression of CPR necessary to detect CYP3A4 activity (Figure 3A). The Vmax value of CYP3A4 activity in the cell homogenates was 30-fold less than that in HLM and Supersomes™ (Figure 3B and Table 1). This is not surprising given that P450s are concentrated in the microsomal fraction and that there may be more CPR and cytochrome b5, which localize to the endoplasmic reticulum (ER) membrane and function as electron suppliers for P450, in HLM and Supersomes™ than in COS-1 cells (Crespi and Miller, 1999). In contrast, the Km value was comparable between the cell homogenates and the other microsomal samples. Expressed CYP3A4 levels were 2–3 times higher with the Bac-Mam system than with the plasmid transfection method, one of the main techniques for heterologous expression (Figure 4A). Hence, the Bac-Mam system is useful for experiments on drug-metabolizing enzymes such as determination of substrate/isoform specificities, and estimation of effects of genetic factors on enzyme activity.

It has been reported that the Bac-Mam system can deliver genes into a wide range of vertebrate cells, some of which show low transfection efficiency when the plasmid transfection with chemical reagent is applied. For example, primary cells including hepatocytes, induced pluripotent stem cells, and neural cells can be transduced by the Bac-Mam virus (Hu, 2005; Makkonen et al., 2015). HepG2 cells are also difficult to be transduced by the plasmid transfection methods. However, the Bac-Mam virus could introduce the CYP3A4 gene into HepG2 cells (Figure 4C), which is consistent with previous studies (Hofmann et al., 1995; Boyce and Bucher, 1996). There was a large difference in the expression of CYP3A4 among the 3 cell lines (Figures 4C,D). As it is known that the CMV promotor is a strong promoter in many cell types, we think that the difference in CYP3A4 expression levels among the 3 cell lines is a result of their sensitivity to infection by baculoviruses. A baculovirus enters mammalian cells via the envelope glycoprotein gp64, and cell surface heparan sulfate proteoglycans and phospholipids have been identified as factors on the mammalian cell side that interact with gp64 (Duisit et al., 1999; Tani et al., 2001). We speculate that quantitative and qualitative differences in these factors among the cells examined in this study led to the differences in CYP3A4 expression levels. In addition, the Bac-Mam system can be applied to large scale expression in mammalian cells which can be grown in suspension culture, including HEK293S GnTI- and CHO cells (Ramos et al., 2002; Morales-Perez et al., 2016). Although it is necessary to optimize the expression conditions in each cell, these findings indicate that the Bac-Mam system is a safe, simple, and efficient approach to analyzing drug-metabolizing enzymes in mammalian cells.

In mammalian cells, it is expected that expressed proteins undergo post-translational modification as they do in the mammalian body, which is a large advantage of a mammalian cell expression system compared to other expression systems with *E. coli*, insect cells, etc. Most UGTs have several *N*-glycosylation sites, Asn-X-Ser/Thr, in their sequence, and glycosylation can affect UGT function (Mackenzie, 1990; Barbier et al., 2000; Nakajima et al., 2010; Nagaoka et al., 2012; Nakamura et al., 2016). Although proteins which have such glycosylation sites undergo modification in insect cells, there is a difference in the glycosylation pattern between mammalian and insect cells (Marchal et al., 2001; Jarvis, 2003). Further, it was reported that UGT activity could be reduced when the enzyme was expressed at high levels with a baculovirus-inset cell system (Oda et al., 2012; Zhang et al., 2012). Taken together, as a next step, we need to analyze UGT using the Bac-mam expression system to confirm if such post-translational modifications are properly performed in this system.

Similar to post-translational modification, protein-protein interactions among drug-metabolizing enzymes can regulate their activities (Backes and Kelley, 2003; Finel and Kurkela, 2008; Ishii et al., 2010). Our team revealed that P450 and UGT also form a complex on the ER membrane and regulate each others function, even though they catalyze far different reactions, and their membrane topologies are opposite (Ishii et al., 2010; Miyauchi et al., 2021). Our previous study indicated that coexpression of P450 and UGT in the same membrane is necessary to see the effects of P450-UGT interactions (Miyauchi et al., 2019). In general, it is difficult to control the expression levels of several target proteins when they were coexpressed using a single expression system. The Bac-Mam system can be easily combined with other methods such as plasmid-transfection, which may aid in the controlled expression of several proteins and permit a more rigorous investigation of protein-protein interactions including those between P450 and UGT.

In conclusion, this study highlights the Bac-Mam system as a convenient approach to analyze drug-metabolizing enzymes in mammalian cells. The Bac-Mam system is safe, simple, and efficient compared to other widely used methods. Hiratsuka’s group recently reported their application of a HEK293FT expression system for heterologous expression of several P450 isoforms, which is also a simple and useful approach for estimating P450 function (Kumondai et al., 2020). These methodological advances are an immense aid in analyzing how genetic- and post-translational-factors affect functions of drug-metabolizing enzymes that result in large inter-individual differences in drug metabolism.

## Data Availability

The original contributions presented in the study are included in the article/Supplementary Material, further inquiries can be directed to the corresponding authors.
