# Supplementary material for: Use of a Baculovirus-Mammalian Cell Expression-System for Expression of Drug-Metabolizing Enzymes: Optimization of Infection With a Focus on Cytochrome P450 3A4
Source: Front Pharmacol. 2022 Feb 22;13:832931. doi: 10.3389/fphar.2022.832931 (PMC8919721; doi:10.3389/fphar.2022.832931)
Supplement: Supplementary file 1 [file Presentation1.PPTX]

## Slide 1
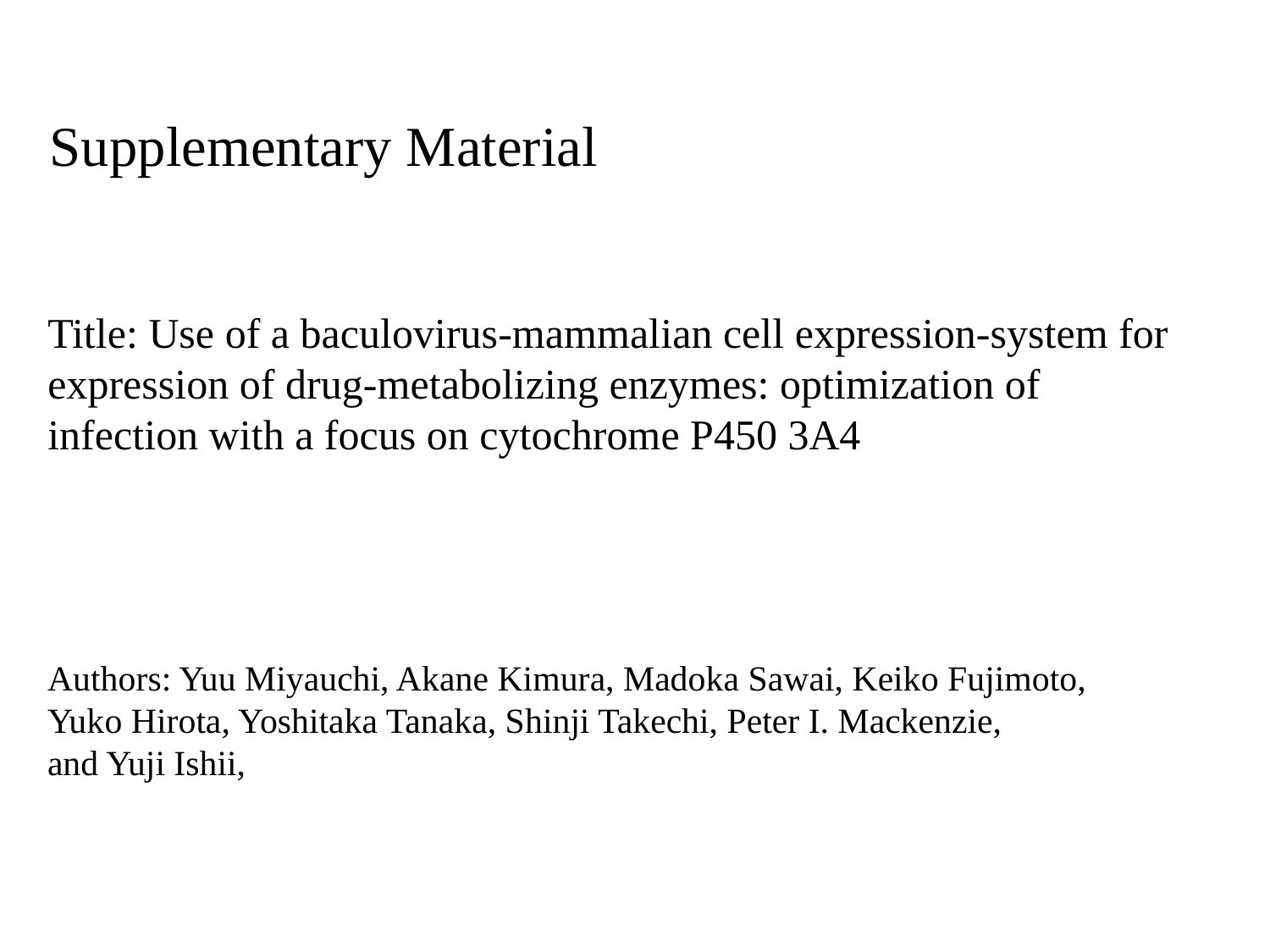

Supplementary Material
Title: Use of a baculovirus-mammalian cell expression-system for expression of drug-metabolizing enzymes: optimization of infection with a focus on cytochrome P450 3A4
Authors: Yuu Miyauchi, Akane Kimura, Madoka Sawai, Keiko Fujimoto, Yuko Hirota, Yoshitaka Tanaka, Shinji Takechi, Peter I. Mackenzie,
and Yuji Ishii,

## Slide 2
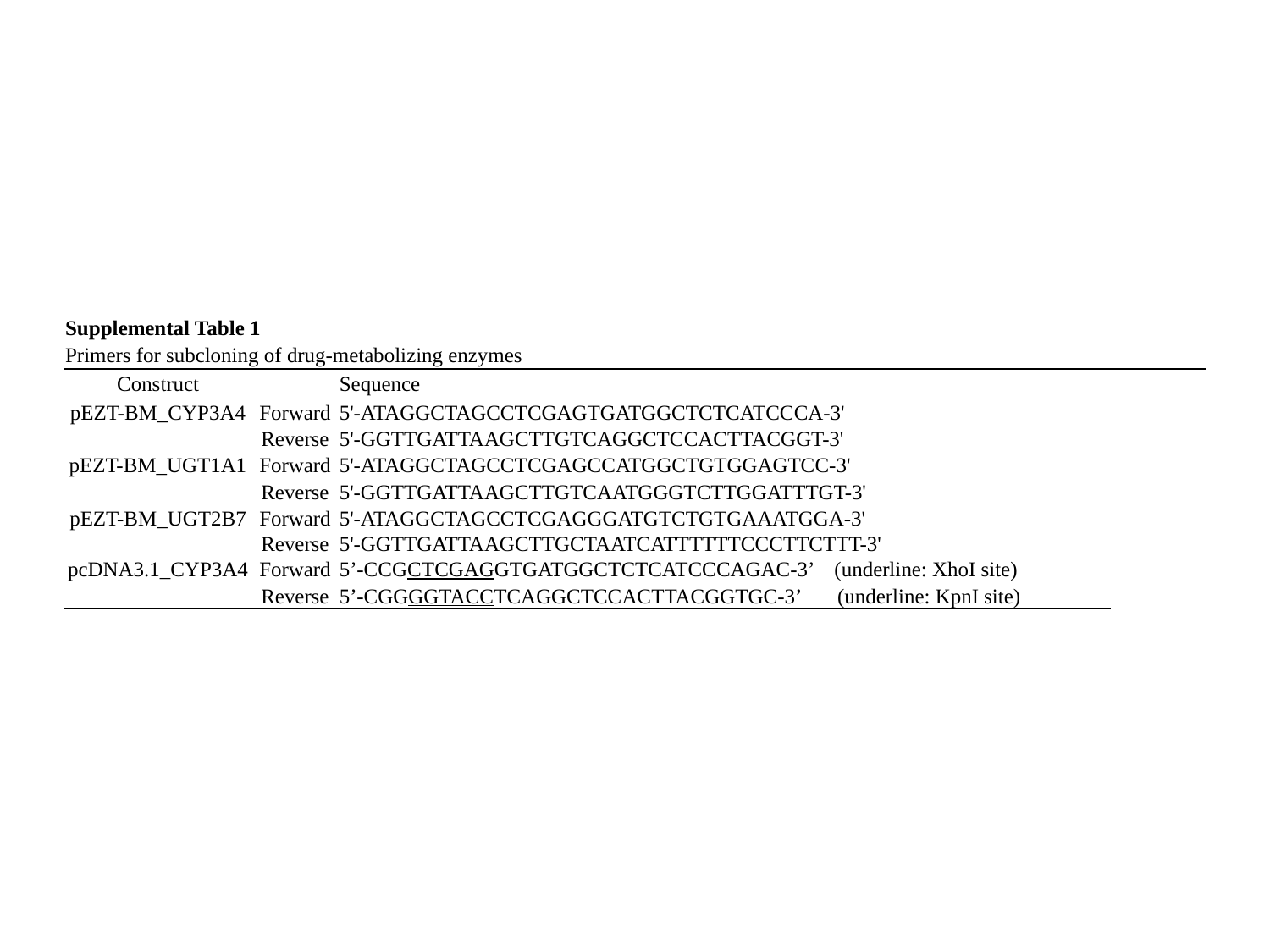

| Supplemental Table 1 | | | |
| --- | --- | --- | --- |
| Primers for subcloning of drug-metabolizing enzymes | | | |
| Construct | | Sequence | |
| pEZT-BM\_CYP3A4 | Forward | 5'-ATAGGCTAGCCTCGAGTGATGGCTCTCATCCCA-3' | |
| | Reverse | 5'-GGTTGATTAAGCTTGTCAGGCTCCACTTACGGT-3' | |
| pEZT-BM\_UGT1A1 | Forward | 5'-ATAGGCTAGCCTCGAGCCATGGCTGTGGAGTCC-3' | |
| | Reverse | 5'-GGTTGATTAAGCTTGTCAATGGGTCTTGGATTTGT-3' | |
| pEZT-BM\_UGT2B7 | Forward | 5'-ATAGGCTAGCCTCGAGGGATGTCTGTGAAATGGA-3' | |
| | Reverse | 5'-GGTTGATTAAGCTTGCTAATCATTTTTTCCCTTCTTT-3' | |
| pcDNA3.1\_CYP3A4 | Forward | 5’-CCGCTCGAGGTGATGGCTCTCATCCCAGAC-3’ (underline: XhoI site) | |
| | Reverse | 5’-CGGGGTACCTCAGGCTCCACTTACGGTGC-3’ (underline: KpnI site) | |
| | | | |

## Slide 3
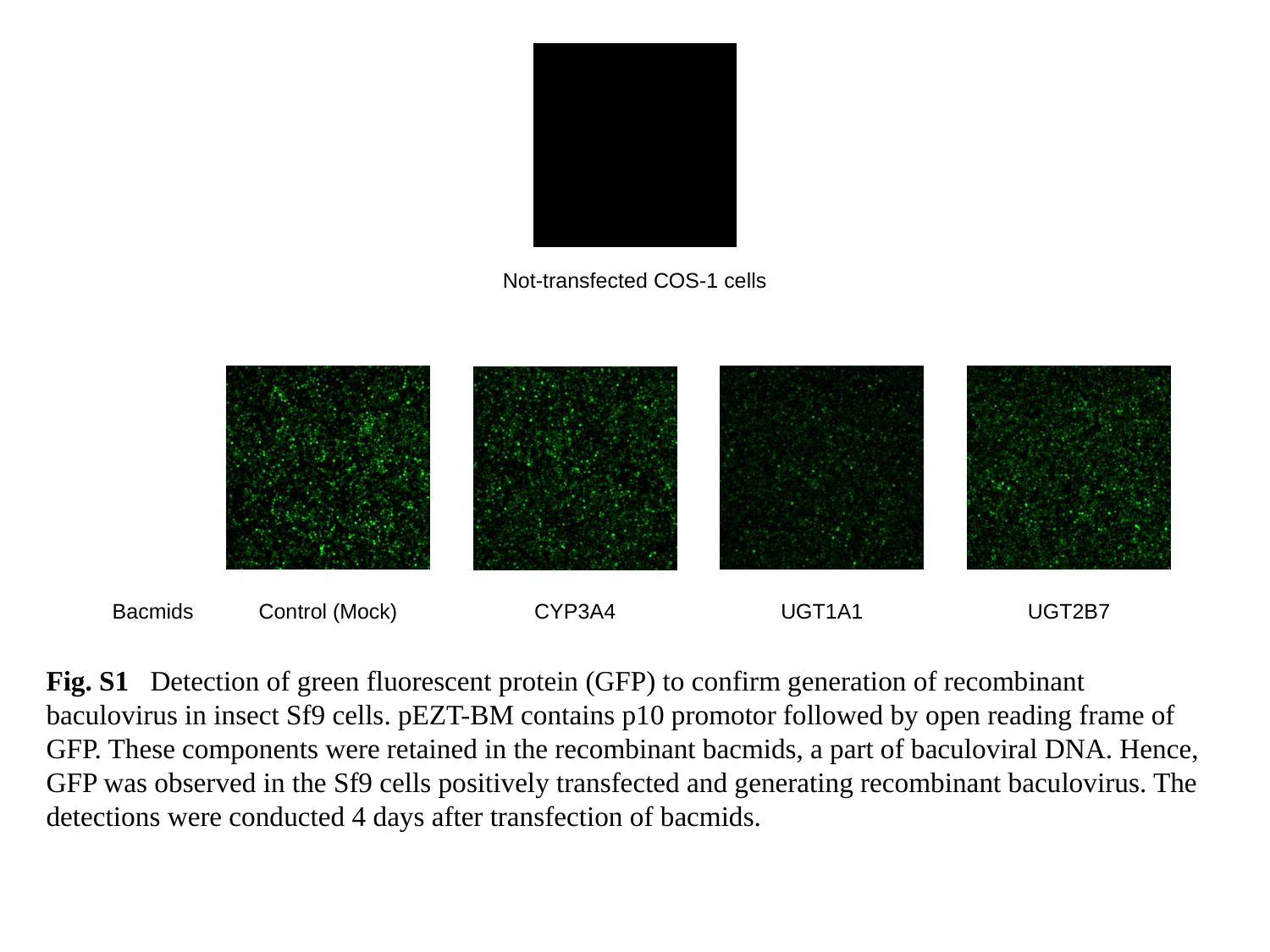

Not-transfected COS-1 cells
Bacmids
Control (Mock)
CYP3A4
UGT1A1
UGT2B7
Fig. S1 Detection of green fluorescent protein (GFP) to confirm generation of recombinant baculovirus in insect Sf9 cells. pEZT-BM contains p10 promotor followed by open reading frame of GFP. These components were retained in the recombinant bacmids, a part of baculoviral DNA. Hence, GFP was observed in the Sf9 cells positively transfected and generating recombinant baculovirus. The detections were conducted 4 days after transfection of bacmids.
